# Quality of care assessment for small and sick newborns and young infants in Pakistan: findings from a cross-sectional study

**DOI:** 10.1186/s12887-022-03108-5

**Published:** 2022-01-29

**Authors:** Nousheen Akber Pradhan, Sumera Aziz Ali, Sana Roujani, Ammarah Ali, Syed Shujaat Hussain, Samia Rizwan, Shabina Ariff, Sarah Saleem, Sameen Siddiqi

**Affiliations:** 1 Department of Community Health Sciences, Aga Khan University, Karachi, Pakistan; 2 Department of Epidemiology, Columbia University, New York, USA; 3 United Nations International for Children’s Education Fund, Country Office, Islamabad, Pakistan; 4 Department of Paediatrics and Child Health, Aga Khan University, Karachi, Pakistan

**Keywords:** Service readiness, Quality of care, Small and sick newborns and young infants, Facility assessment, Pakistan, Neonatal mortality, and Inpatient care units

## Abstract

**Introduction:**

Pakistan is facing a challenging situation in terms of high newborn mortality rate. Securing pregnancy and delivery care may not bring a substantial reduction in neonatal mortality, unless coupled with the provision of quality inpatient care for small and sick newborns and young infants (NYIs). We undertook this study to assess the availability and quality of newborn care services provided and the readiness of inpatient care for NYIs in Pakistan.

**Methods:**

We conducted a cross-sectional study across Pakistan from February to June 2019, using a purposive sample of 61% (23) of the 38 sick newborn care units at public sector health care facilities providing inpatient care for small and sick NYIs. We interviewed facility managers and health care providers by using structured questionnaires. We observed facility infrastructure and relevant metrics related to the quality of inpatient care such as types of infant care units and essential equipment, drugs, staffing cadre and facility management practices, quality assurance activities, essential services for small and sick NYI care, discharge planning, and support, quality of NYIs care record, and health information system.

**Results:**

Of the 23 facilities assessed, 83% had newborn intensive care units (NICUs), 74% reported Special Care Units (SCUs), and only 44% had Kangaroo Mother Care (KMC) Units. All facilities had at least one paediatrician, 13% had neonatologists and neonatal surgeons each. Around 61 and 13% of the facilities had staff trained in neonatal resuscitation and parental counseling, respectively. About 35% of the facilities monitored nosocomial infection rates, with management and interdisciplinary team meetings reported from 17 and 30% of the facilities respectively preceding the survey. Basic interventions for NYIs were available in 43% of the facilities, only 35% of facilities had system in place to monitor nosocomial infections for NYI care. Most (73%) of reviewed records of NYIs at 1–2 days had information on the birth weight, temperature recording (52%), while only a quarter (25%) of the observed records documented danger signs. Mechanism to support discharge care by having linkages with community workers was present in 13% of the facilities, while only 35% of the facilities have strategies to promote adherence after discharge. Majority (78%) of facilities reported monitoring any newborn/ neonatal care indicators, while none of the sub-units within facilities had consolidated information on stillbirths and neonatal deaths.

**Conclusion:**

The study has demonstrated important gaps in the quality of small and sick NYI inpatient care in the country. To avert neonatal mortality in the country, provincial and district governments have to take actions in improving the quality of inpatient care.

## Introduction

Among under-five deaths, 2.4 million newborns die within the first month of their birth, accounting for 47% of the global burden of under-five mortality [1]. Pakistan bears a significant burden of newborn deaths. The country was unable to reach the Millennium Development Goal of reducing child mortality, and has been considered one of the top 10 countries with highest newborn mortality rate, where for every 1000 newborns, 42 die before the first month of their life [2]. Although the Government of Pakistan (GoP) with the support of many development partners has made efforts to improve maternal and child health by increasing access to antenatal care, facility-based delivery, and strengthening district health systems, these efforts have not made a significant dent in reducing neonatal mortality [3]. The Sustainable Development Goals provide a renewed opportunity to achieve the global target of 12/1000 neonatal deaths, as the GoP aims to achieve universal health coverage by 2030 [4].

Increasing access alone is unlikely to improve neonatal outcomes unless coupled with efforts to improve the quality of inpatient care for small and sick newborns and young infants (NYIs) [5–8].

Several studies have consistently recognized that the quality of neonatal inpatient care is a major concern [7, 9]. Improving access and quality of inpatient care is crucial to achieving the objectives of the Every Newborn Action Plan (ENAP) of ≤10 newborn deaths per 1000 live births by 2035 [10]. Numerous bottlenecks exist in LMICs such as inadequate infrastructure, scarcity of health workforce, inadequate budget, and lack of appropriate payment mechanisms [11]. These factors greatly affect the quality of care for NYIs that required specialized care at inpatient care settings in resource constrained settings.

Development partners such as the United Nations International Children’s Education Fund (UNICEF) has been actively supporting the GoP in its endeavors to reduce neonatal mortality by establishing district-level Sick Newborn Care Units (SNCUs) across the country and building the capacity of staff in essential care for small and sick NYI care [12].

Alongside strengthening inpatient care services for small and sick NYIs, there is also a need to strengthen community-based interventions. Simple and low-cost interventions to prevent infections in neonates have been documented to reduce 23% risk of neonatal mortality [13, 14].

Realizing the slow progress in reducing in newborn mortality rate in Pakistan in last few decades and the country being regarded as the riskiest place to be born [15], this demands a holistic assessment of the existing health care system at the inpatient care units in public sector hospitals. Therefore, the Aga Khan University (AKU), Pakistan working in partnership with UNICEF conducted a study to assess the readiness and quality of inpatient care for small and sick NYIs provided in public sector hospitals across the country. The objectives include assessing the readiness of health care service provision for small and sick NYIs at public sector health care facilities in Pakistan for the; (1) availability of infrastructural support, equipment, and essential drugs, (2) availability of trained staff and capacity building needs of the health workforce, (3) facility management and quality assurance practices at SNCUs, (4) availability of interventions for NYI care, (5) quality of NYIs records to capture the essential parameters related to the care of small and sick NYIs, (6) health information system at public sector health care facilities, and (7) discharge planning and support.

## Material and methods

UNICEF in partnership with the GoP has supported strengthening of 38 SNCUs in public sector hospitals across all four provinces (Sindh, Punjab, Baluchistan, and Khyber Pakhtunkhwa (KPK)), the federal capital- Islamabad Capital Territory (ICT), and two administrative regions (Azad Jammu & Kashmir (AJK) and Gilgit–Baltistan (GB)) of Pakistan. We conducted a cross-sectional study during the year 2019 in 23 [61%] of these public sector healthcare facilities to assess inpatient care for NYIs (0–59 days of age). All facilities were purposively selected in consultation with the UNICEF country office, the Ministry of National Health Services Regulation & Coordination, and the provincial health departments. The eligibility was contingent on distribution across all provinces and regions and provision of round-the-clock inpatient care for NYIs at these facilities. The selected facilities were ensured to provide a good representation from better and worse performing regions in terms of neonatal mortality across all geographies in the country.

Data was collected using a set of structured questionnaires and survey tools designed by UNICEF for a multi-country study on the same subject [16]. These tools were pre-tested in a non-study facility to assess the flow, coherence, and sequence of questions, and required amendments were made. Two data collection teams, each with one paediatrician and two doctors were trained over 1 week on the data collection tools. These teams surveyed sampled health care facilities and were closely supervised and supported in the field by the principal investigator and co-investigator.

The data collection elements included interviews with health care providers and direct observations of the services provided. The eligibility criteria of the facilities include the public sector health care facilities with 24/7 inpatient care units for small and sick NYIs. After receiving written informed consent from the in charge (supervisors and managers) of the eligible facilities, the study team familiar with the national (Urdu) or local language, administered the questionnaire to the facility in charge and service providers who were actively engaged in the provision of care to NYIs.

The structure of the facility assessment tool was based on health system components/building blocks defined by the World Health Organization (WHO) [8]. Processes of care at the facilities were assessed using eight domains listed in the WHO maternal and newborn health quality of care framework [15]. The UNICEF data collection tools used for this study broadly covers all eight domains in general. The findings from this paper mainly report standards 1, 2, 7 and 8 which relates to women and newborn receiving care and management of complications, health information system, required health workforce, and availability of appropriate infrastructure, medicines, supplies, equipment for routine maternal and newborn care at facilities respectively [17]. The indicators within these domains were selected by UNICEF for the multi-country study.

Direct observations included assessment of facility infrastructure and relevant metrics related to the quality of inpatient care, staffing, availability and functionality of equipment, facility management practices, quality assurance activities, essential services and interventions for NYI care, and discharge planning and support. Within infrastructure, we assessed three levels of inpatient care for newborns as defined by WHO [17]. These are described below.

Level one is basic care. This is also called KMC that offer beds for the mother or another person to comfortably provide skin-to-skin care and specific assistance (if needed) for infant to receive breast milk (either through breastfeeding or alternative methods) [8]. Level two is Special Care. As defined by UNICEF, this is referred as SNCUs. At this level, various levels of interventions and treatment for sick infants are provided. If a facility has a NICU, the SNCU usually cares for moderately ill infants providing treatments, but not at the level offered in NICUs, and not with the intensive monitoring expected in NICU. Level three is Neonatal Intensive Care. We assessed availability of NICUs that provide close monitoring (e.g., blood transfusions, incubator, or radiant warmer care) of newborns in critical condition. A neonatologist is ideally available 24 h in NICUs.

Essential services and interventions assessed for small and sick NYIs care for UNICEF multi-country study are listed in Table 1. Also, we explored management and supervision practices and quality of care attributes across all facilities.

**Table 1:** Assessment criteria of essential services and interventions for small and sick newborns and young infants at inpatient care settings

**1. Basic interventions for newborn and young infants**	
▪ Provide thermal management	
▪ Provide feeding and lactation support	
▪ Ensure practice of handwashing between infants	
▪ Ensure safe practice of keeping one infant in a cot	
**2. Interventions for sick newborns**	
▪ Administer oxygen	
▪ Provide intravenous fluids	
▪ Provide alternatives to breastfeeding	
▪ Exchange transfusion	
**3. Diagnostic and treatment services for neonatal conditions**	
▪ Diagnose possible neonatal sepsis/ severe bacterial infection	
▪ Provide antibiotics for neonatal infections	
▪ Diagnose neonatal respiratory distress/disorders	
▪ Treat neonatal respiratory distress	
▪ Detect and manage hypothermia	
▪ Detect hyperbilirubinemia	
▪ Manage hyperbilirubinemia	
▪ Diagnose / investigate cause of seizures	
▪ Treat seizures	
**4. Routine newborn screening for congenital birth defects**	
▪ Assess congenital birth defects	
▪ Conduct surgical repair for any congenital defects	
▪ Conduct routine blood test for congenital disorders such as hypothyroidism, phenylketonuria, cystic fibrosis etc.	
▪ Assess of newborn hearing	
▪ Assess retinopathy	

The table presents the assessment criteria for basic interventions, intervention for sick newborns, and diagnosis and treatment of common neonatal conditions and routine newborn screening for congenital birth defects as defined by UNICEF

A total of 184 medical records of small and sick inpatient NYIs of 0–59 days were reviewed. The criterion was to randomly review five records per facility for sick children that were in their (1) 1–2 days of birth and admission at inpatient care unit, and (2) > 3rd day of receiving care at the facility. The purpose of enrolling NYIs with 1–2 days of birth and admission at inpatient care units was to assess the documentation of birth weight, Appearance, Pulse, Grimace, Activity, and Respiration (APGAR), temperature, danger signs, and congenital anomalies. On the other hand, the record review of NYIs in their >3rd or more days was carried out to get adequate information on inpatient medical records to determine the pattern and quality of routine monitoring of the newborns at birth that included NYIs’ age, medical history, diagnosis, mode of delivery and note on transfer and referral. The documentation related to the number of live births, perinatal deaths, neonatal deaths, and average bed occupancy were collected for the previous 3 months. Lastly, the research team assessed health workers’ qualifications, working conditions and experience, and their training in related specialties.

### Analysis

The data was entered into Statistical Package for Social Sciences, (SPSS) (version 19.0) under the supervision of a data manager. After data cleaning, descriptive statistics, frequencies and proportions were estimated to determine characteristics under investigation. Service readiness analyses covered domains such as type of infant care units, availability of essential equipment and drugs, staffing cadre, training of staff and facility management practices, quality assurance activities related to small and sick NYIs, availability of interventions for NYI care, discharge planning and support, quality of NYIs care and health information system.

## Results

The results were analysed and are presented under the following domains: (i) types of NYIs care units and availability of essential equipment and drugs; (ii) staffing cadre, training of the staff and facility management practices; (iii) quality assurance practices related to NYIs care; (iv) availability of interventions for NYIs care and discharge planning and support; (v) quality of NYIs records; and (vi) health information systems.

### Type of NYIs care units and availability of essential equipment and drugs

The distribution and levels of hospitals across all regions are depicted in Table 2. Of the total 23 facilities assessed, 83% had SNCUs, 74% had NICUs, and 44% had KMC Units (Table 3).

**Table 2:** Distribution and level of hospitals assessed across provinces and regions of Pakistan

S.No	Provinces and administrative regions	National referral hospital^a^	Provincial referral hospitals^b^	District hospitals^c^	Tertiary care hospitals^**d**^	Total
1.	Punjab	–	1	5	–	6
2.	Sindh	–	1	1	2	4
3.	Khyber Pakhtunkhwa	–	1	4	–	5
4.	Baluchistan	–	2	2	–	4
5.	Islamabad Capital Territory	1	–	–	–	1
6.	Azad Kashmir	–	–	2	–	2
7.	Gilgit-Baltistan	–	1	–	–	1
Total		1	6	14	2	23

^a^This is a referral hospital with basic intensive care facilities, and the highest-level health facility in the country. Referral hospitals also often serve as teaching hospitals, having the highest expertise and offer the most diverse services. Their target population is the entire country. Lower level hospitals refer patients to the national referral hospital

^b^A provincial/regional referral hospital often receives referrals from lower level hospitals (i.e., district hospitals) and other facilities, usually within the same region. A regional referral hospital can usually perform common major surgeries and also has some specialist physicians

^c^A district hospital may be a referral hospital for lower level hospitals or may be the first level of hospitals. District hospitals usually provide basic level surgical and medical inpatient services and can perform basic diagnostic tests and treatment. A district hospital is linked in the government health system, whether it is managed by the government or by another authority

^d^A tertiary referral hospital is a hospital that provides tertiary care; a level of health care obtained from specialists in a large hospital after referral from the providers of primary and secondary care. In context of this study, tertiary care teaching hospitals were selected that partners with medical and nursing schools, education programs and research centers to improve health care through learning and research

**Table 3:** Availability of infrastructure and qualified staff for neonatal care (*n* = 23)

S. No.	Level of facility	Number of facilities surveyed	Newborn and young infant care units			Availability of qualified staff			
			NICUs	SCUs	BCU/ KMC	Paediatrician	Neonatologist	Neonatal Surgeon	Neonatal Nurse Specialist
1.	National referral hospital	1	1	1	1	1	1	1	0
2.	Provincial referral hospital	6	4	5	2	6	1	1	0
3.	District hospital	14	13	9	6	14	1	0	2
4.	Tertiary care hospital	2	1	2	1	2	0	1	0
**Total (percent)**		**23**	**19 (83%)**	**17** **(74%)**	**10** **(44%)**	**23** **(100%)**	**3** **(13%)**	**3 (13%)**	**2 (9%)**

There were variations observed among provinces/federal territories as regards the availability of different types of infant care units. All facilities in AJK, ICT, and Punjab; 60% in KPK; and 50% of the facilities in Sindh and Baluchistan were equipped with NICUs, while GB did not have the same type of unit. The availability of KMC was reported from the only surveyed facility in ICT and most (83.3%) of facilities in Punjab, while none of the surveyed facilities in the federal territories and KPK had KMC units. However, a quarter of the facilities in Sindh and half of the facilities in Baluchistan were equipped with KMC units.

At KMC units, less than a quarter (22%) of facilities reported availability of job aid/ guidelines related to KMC, intermittent KMC (skin-to-skin care for newborns), and provision of infant clothing. The policy to encourage 24-h parental access to infant was however, reported from only 2 out of 23 surveyed facilities (8.7%).

The functionality of all three units was also gauged during field site observations. Our observations were in accordance with the objectives specified. The majority of the surveyed facilities were equipped with the three essential equipment required for the inpatient care of NYIs. The availability of phototherapy lights was reported from all surveyed care facilities. Majority (96%) of the facilities were also well equipped with radiant warmers and incubators.

The practice for preventive and corrective maintenance for equipment was notified from less than half of the facilities (43%). Majority (91%) of the facilities reported functional phototherapy lights and incubators, whereas, the functionality of radiant warmer was reported from 70% of the facilities. The availability and functionality of the equipment and functional status were reported by the staff on duty.

Altogether, 21 essential drugs (in seven categories) for the treatment of small and sick NYIs were assessed across all surveyed facilities. This includes antimicrobials, neurologic, inotropic, bronchodilators, corticosteroids, vitamin K, and IV infusions. None of the facilities reported having all 21 drugs at the time of assessment. Among antimicrobials, the first line of drugs such as ampicillin and gentamycin were available in only 45 and 54% of the facilities respectively. Under neurologic drugs, the most essential ones such as phenobarbital injection were missing from all 23 health care facilities and the phenytoin injection was only available in a few (23%) of the facilities. Most (86%) of the facilities were observed to have one of the two steroids. The inotropic drug was available in 68% of the facilities. The availability of bronchodilators (theophylline/aminophylline injections) was reported from the majority (68%) of the facilities. Vitamin K injection was found in 64% of the facilities. The availability of four IV infusions across overall facilities ranged from 59% to 95%. Provincial variations were noted for the availability of essential drugs. Essential antimicrobial was found 100% in ICT, 75% of the facilities in Punjab, and 20% in Punjab, while none of the facilities in GB had it and 50% of the facilities in KPK had one of the two essential antimicrobials. Of the seven neurologic drugs, none of the regions were found to have the availability of two essential ones. Facilities in the ICT and GB region reported having one out of two, while it was reported to be missing from Sindh and Punjab regions. Availability of four IV infusions was found in ICT, AJK, and GB regions, while other regions reported some variations.

### Staffing cadre, training of the staff, and facility management practices

All 23 facilities reported having at least one paediatrician. Only 13% of the facilities had neonatologists and neonatal surgeon. Whereas, only few (9%) of the facilities reported having neonatal nurse specialists, as illustrated in Table 2. Among all provinces and regions, only AJK had a neonatal nurse specialist, while the availability of neonatal surgeons and a paediatric retinal specialist was reported from few (13%) facilities in Sindh and Baluchistan.

**Fig. 1:**
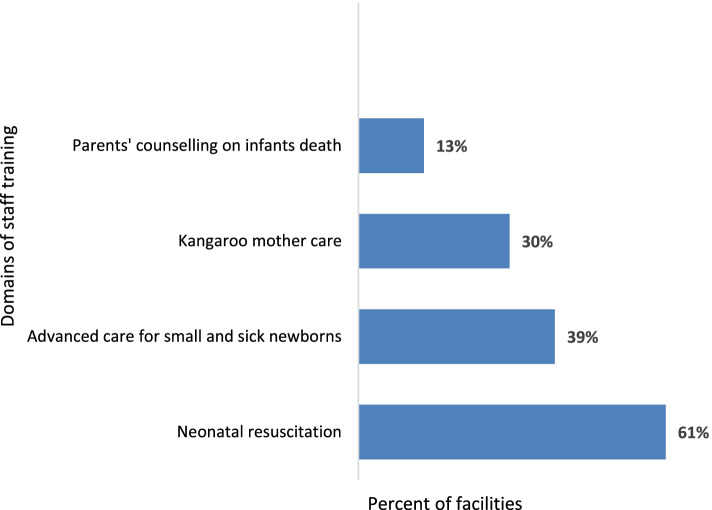
Percent of facilities reported to conduct staff training related to newborn and young infant care in last 24 months (*n* = 23). It shows findings reported from 23 inpatient care facilities in Pakistan on staff training related to NYIs neonatal resuscitation, advanced care for small and sick newborns, parents’ counselling on infant deaths and kangaroo mother care

Figure 1 depicts capacity-building initiatives for the staff (undertaken by the facilities in last 24 months preceding the survey) in the assessed health care facilities in four domains for NYI care: (1) kangaroo mother care, (2) neonatal resuscitation, (3) advanced care for newborn, and (4) parental counselling on infant deaths. Of the four domains, 61% of the facilities had trained staff in neonatal resuscitation. For the remaining three domains, we found that less than 50% of the staff got trained in advanced care for small and sick NYIs (39%), kangaroo mother care (30%), and parental counselling on infant death (13%).

External supervision for improved facility management during the last 3 months was reported by 39% of all facilities (Table 4). In context of this study, external supervision was considered by the supervisory visits paid by District Health Management and/ Provincial Health Management representative to ensure smooth adherence of standards being followed at the facility level [16].

**Table 4:** Facility Management and Quality Assurance Practices at Sick Newborn Care Units (*n* = 23)

S. No.	Level of facility	Number of facilities surveyed	Management and Supervision				Quality Assurance Practices			
			External supervision^a^	Management team meetings^b^	Interdisciplinary team meetings^c^	Budgetary flexibility^d^	Monitoring quality of care indicators^e^	Monitoring nosocomial infection^f^	Accreditation/ certification^g^	Designated Baby-friendly facility^h^
1.	National referral hospital	1	0	0	0	1	1	1	0	1
2.	Provincial referral hospital	6	3	2	2	3	4	2	0	3
3.	District level hospital	14	5	1	5	8	10	5	5	3
4.	Tertiary care hospital	2	1	1	0	1	1	0	1	2
Total (percent distribution)		23	9 (39%)	4 (17%)	7 (30%)	13 (57%)	16 (70%)	8 (35%)	6 (26%)	9 (39%)

The table presents findings on four key practices related to management and supervision, alongside four key quality of care attributes for NYIs across all 23 surveyed facilities at four different levels of hospitals in Pakistan

^a^External supervision is usually conducted by person who work for a managing authority or a technical service sector

^b^Identification of issues requiring intervention and information sharing to support quality services usually requires some type of meeting. Management team meetings are the common strategy for routinely bringing together the relevant persons/departments for NYIs care. These teams may address administrative issues, and also may address the systems and how they are functioning to maintain and improve services for NYIs. We assessed whether management team meeting are carried out that address small and sick NYIs cases at inpatient care settings and whether those meetings were held once every quarter

^c^For complex patients, such as sick NYIs, multiple specialist services or disciplines (interdisciplinary) may be needed to improve the immediate outcome as well as the long-term prognosis for the patient. Interdisciplinary team meetings are expected to improve coordination, identification of needs, and to result in better planning and smoother teamwork for any particular patient

^d^Budgetary flexibility is the autonomy exercised by the facility over financial matters

^e^Indicators related to quality of care for small and sick NYIs includes perinatal mortality rate, neonatal mortality rate, and case fatality rate

^f^Any system in place at inpatient settings to monitor nosocomial infections

^g^Facilities participating in any accreditation and/ certification program

^h^This involves 10 criteria for Baby-Friendly Hospital Initiative Accreditation (2018) by WHO and UNICEF; (1) compliance with the international code of marketing of breastmilk substitutes, (2) sufficient knowledge, competence and skills of staff, (3) discuss importance of breastfeeding to pregnant women and families, (4) facilitate immediate and uninterrupted skin-to-skin contact and support mothers to initiate breastfeeding as soon as possible after birth, (5) support mothers to initiate and maintain breastfeeding and manage common difficulties, (6) do not provide breastfed newborns any food or fluids other than breast milk, unless medically indicated, (7) enable mothers and their infants to remain together and to practise rooming-in 24 h a day, (8) support mothers to recognize and respond to their infants’ cues for feeding, (9) counsel mothers on the use and risks of feeding bottles, teats and pacifiers, and (10) coordinate discharge so that parents and their infants have timely access to ongoing support and care

There were 17%, 4 of the 23 facilities assessed that conducted management team meetings. The practice of inter-disciplinary team meetings was reported by 30% of management personnel. This was mostly being practiced in AJK and Punjab. Lastly, the assessment showed that more than half (57%) of the facilities reported control over budgetary matters (Table 4).

### Quality assurance practices related to NYIs care

All 23 facilities were assessed for four dimensions of quality of care: (1) monitoring quality of care indicators specific to NYI care, (2) monitoring of nosocomial infections, (3) accreditation/ certification, and (4) designated baby-friendly hospitals (Table 4).

Overall, 70% of the facilities reported monitoring any quality care indicators for performance measurement. Only, 35% of facilities revealed monitoring of nosocomial infections with variations across provinces. All facilities assessed in AJK, 50% in Punjab, and 40% in KPK had a system in place for the monitoring of nosocomial infections. Less than 30% of the facilities reported participating in an accreditation or certification program (at the time of survey or in the past).

As regards the baby-friendly hospital (in terms of either the certification and external validation of the implementation of policies and practices for the protection, promotion, and breastfeeding support), as defined by WHO/UNICEF Baby-Friendly Hospital Initiative [16], nearly 40% of the facilities were designated as baby-friendly. This includes a facility in ICT and 75% of the surveyed facilities in Baluchistan and Sindh each.

### Availability of interventions for NYIs care and discharge planning and support

**Fig. 2:**
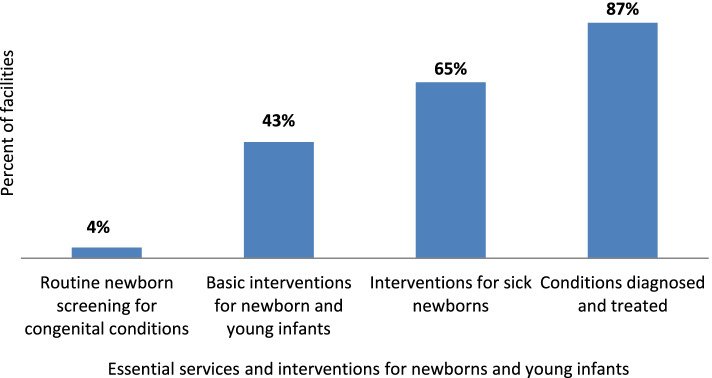
Percent of facilities with essential services and interventions for small and sick NYIs care (*n* = 23). It illustrates the facilities with the provision of four essential services and interventions for NYIs care across all surveyed facilities. This includes routine newborn screening for congenital conditions, basic interventions for NYIs, interventions for sick newborns, and services for the diagnosis and treatment of neonatal conditions

The assessment demonstrated that of the four services and interventions assessed, diagnostic and treatment services for the neonatal conditions were reported from majority (87%) of the inpatient care settings. Basic interventions for sick NYIs care and interventions for sick newborns were reported from 43% and 65% of the facilities respectively. We also assessed essential services to diagnose congenital birth defects; only offered in 4% of facilities. (Fig. 2).

The percentages shown in Fig. 2 represent only those facilities that reported 100% of the services under all four essential services and interventions for NYIs care (highlighted in Table 1).

Around 52% of the surveyed facilities reported a system or guidelines for the discharge plan for home care of the NYIs. More than one-third (35%) of the surveyed facilities reported providing strategies for promoting adherence after infant discharge (Table 5). A system to support home care for NYIs after discharge via community health workers was reported by only 13% of the facilities.

**Table 5:** Discharge planning, patient support and case reviews for neonatal care (*n* = 23)

S.No	Level of facility	Number of facilities surveyed	Discharge planning and patient support			Case reviews		
			System for discharge planning	Linkages with CBWs	Strategies to promote adherence after discharge	Patient case review^a^	Near miss events^b^	Perinatal or neonatal death reviews^c^
1.	National referral hospital	1	0	0	1	1	0	1
2.	Provincial referral hospital	6	3	0	3	5	3	1
3.	District level hospital	14	9	3	4	8	5	6
4.	Tertiary care hospital	2	0	0	0	2	1	2
**Total (percent)**		**23**	**12 (52%)**	**3 (13%)**	**8 (35%)**	**16 (70%)**	**9 (39%)**	**10 (43%)**

^a^A case review is a formal meeting where information about a current or discharged patient is presented, usually by the primary physician for that patient, and issues related to diagnosing, treating, and improving the outcome are discussed

^b^Near miss events relates to newborns that almost died at birth

^c^Perinatal death reviews include meetings where information about stillbirths and infants born alive but who died within 7 days is gathered and presented

Variations were reported among provinces with regards to the discharge planning for NYI care. Both surveyed facilities in AJK, 75% in Baluchistan, 50% in Sindh, and 40% in KPK had a system in place for discharge planning. A similar system was found to be lacking in GB. All the surveyed facilities in ICT and AJK reported having strategies for promoting adherence after infant discharge; however, one facility in Baluchistan (1/4), 2/5 in KPK, 2/6 in Punjab reported strategies for promoting adherence after infant discharge. Facilities in Sindh and GB lacked attention on post-discharge care for infants.

### Quality of NYIs records

A total of 184 records of NYIs at 1–2 days (*n* = 79) and > 3–59 days (*n* = 105) were reviewed to determine routine monitoring of NYIs and information recorded for assessing quality and planning for discharge. Some of the essential parameters assessed in the reviewed records include a note on birth weight, vital signs, APGAR scores, and congenital anomalies. The documentation was considered as a proxy indicator for the assessment of NYIs on these essential parameters by the attending health care provider. Most (73%) of reviewed records of NYIs at 1–2 days had information on the birth weight, temperature recording (52%), while only a quarter (25%) of the observed records documented danger signs. Also, the documentation of the congenital anomalies and APGAR scores were observed in one-third of the reviewed records (Table 6).

**Table 6:** Review of newborn and young infants’ records at sick newborn care units (*n* = 23)

S. No	Level of facility	Number of records reviewed	Documentation of newborns’ assessment parameters (*n* = 79)							Documentation for of admission history and physical examination upon arrival of newborn (*n* = 105)				
			Birth weight	Danger signs	Temperature	Congenital abnormalities	Apgar score	Number of records reviewed	Infant age	Transfer/referral note	Diagnosis/symptoms	Patient history	Pregnancy history	Mode of delivery
1.	National Referral Hospital	**5**	5	5	5	5	4	**5**	5	5	5	5	5	5
2.	Provincial Referral Hospital	**20**	18	6	10	7	12	**34**	34	18	30	27	27	27
3.	District Hospital	**45**	26	7	18	10	7	**56**	53	27	52	46	14	38
4.	Tertiary Care Hospital	**9**	9	2	8	0	5	**10**	9	10	8	5	5	5
Total (percent)		**79**	**58 (73%)**	**20 (25%)**	**41 (52%)**	**22 (28%)**	**28 (35%)**	**105**	**101 (96%)**	**60 (57%)**	**95 (91%)**	**83 (79%)**	**51 (49%)**	**75 (71%)**

Of the 105 records of NYIs (> 3–59 days old) reviewed for the documentation related to clinical examination at the time of admission, the majority (96%) of records had the information on infants’ age; 79% on patient’s history and 49% had a note of pregnancy history. Over 70% of records had also captured information on the mode of delivery. Overall, 57% of reviewed records captured information on transfer/referral notes from the referring unit at the time of admission (Table 6).

### Health information system

Assessment of facility practices for collating and monitoring information showed that 83% of the surveyed facilities routinely submitted electronic or paper-based information to an external body, such as to the directorate of health services or a regional technical department. Only 9% of the surveyed facilities reported compiling the reports without submission to higher levels. In addition, 87% of the surveyed facilities reported submitting birth information to the National Vital Statistics. Regarding newborn health monitoring, 78% of the facilities reported monitoring any newborn/ neonatal care indicators. Almost all facilities reported having data accessible for neonatal deaths and stillbirths in separate units, however, none of the sub-units within facilities had consolidated information.

## Discussion

Newborn survival has become a challenge in Pakistan. Of every 1000 babies born, 42 die before the end of their first month [2]. This situation is unacceptable and requires a multipronged approach if the numbers must improve. One such intervention is to ensure the availability of and access to good quality inpatient care for NYIs, especially in secondary and rural hospitals of LMICs such as Pakistan. The country has over 700 large and small public sector hospitals. Although the study was carried out on a small sample of 23 public hospitals, it is illustrative of the prevailing issues surrounding NYIs care in the country. This study was undertaken principally to assess the availability, quality, and readiness of services, and inpatient care for NYIs in multiple hospitals of the country and is part of a larger multicounty initiative by UNICEF. There is a National Integrated Reproductive Maternal and Neonatal Health Strategy (IRMNCH) (2016–2020) that emphasizes staff training, supervision, referral system, and quality care for newborn services in general [18]. Every province has also defined its service delivery standards for hospitals, including physical infrastructure, human resources, equipment, supplies and drugs, service package, information system, etc., at district and taluka headquarter hospitals. The availability of three levels of infant care units at different levels of hospitals has not been explicitly stated in IRMNCH strategy, however, inpatient paediatric care has been specified for all categories of hospitals with slight variations in the number of staffing requirements and equipment [19]. Therefore, the quality standards assessed in this study conform to the standards defined by UNICEF for small and sick newborns, WHO standards for newborn care in health facilities [17, 20], and also reflect the provincial standards for health service delivery at hospitals [19].

The study found that infant care units such as NICUs and SNCUs were generally available in the surveyed facilities. However, the availability of KMC units remained low across the district and provincial hospitals, indicating important gaps in the provision of essential newborn care services at these facilities. The provision of KMC services has been one of the important interventions by UNICEF. However, our results revealed the availability of KMC units in less than 50% of surveyed facilities.

Policymakers, researchers, and key stakeholders in Pakistan have highlighted KMC as an essential component to save newborn lives [21]. This has not been translated into functioning KMC units at health care facilities. As our findings showed that less than a quarter of the facilities offered skin-to-skin care and provided breastfeeding support. Given the evidence to date, KMC units are significant in reducing neonatal mortality and have been identified as a crucial element of newborn health initiatives by ENAP [22]. A meta-analysis of KMC and neonatal outcomes among low birth weight newborns, compared to conventional care KMC was associated with 36% lower mortality (RR 0.64; 95% [CI] 0.46, 0.89), thereby warranting interventions for its scale-up [23]. KMC is a cornerstone of facility-based care for small and sick NYIs [24] and has been endorsed by WHO for the care of low birth weight children as a complement to conventional neonatal care [25]. Therefore, KMC units can potentially be integrated into SNCUs and NICUs to improve neonatal health outcomes in Pakistan.

Alongside infrastructural support, the availability of essential equipment is also significant to improve the quality of NYIs care. Our findings reveal that most of the facilities were equipped with the necessary equipment. Three necessary equipment (radiant warmers, phototherapy lights, and incubators) assessed in the surveyed health care are in accordance with the minimum level health service delivery standards for hospitals in Pakistan [19, 26, 27].

This showed good progress in the available stock of required supplies and equipment, as earlier most of the neonatal care settings in the government hospitals of Pakistan were not sufficiently equipped [28]. The gap, however, remains about the preventive and corrective maintenance of equipment, where less than half of facilities reported carrying out preventive and corrective maintenance of equipment. Such findings are comparable with a study conducted in Ethiopia, which showed that almost all of the facilities providing newborn care were not supported by biomedical engineers [29]; required for the preventive and corrective maintenance of the equipment.

Availability of essential drugs is also crucial to saving the lives of small and sick NYIs in inpatient care settings. Our study reported inadequate availability and variations in the availability of essential drugs across different regions. The shortcoming in the availability of drugs for newborns has also been reported from studies in Kenya [30] and India in public sector hospitals [31].

Adequate stock and skill mix of the health workforce to provide neonatal services is of paramount importance towards improving the quality of NYIs care. The available provincial standards mandate at least one paediatrician at an inpatient care setting and a maximum of 3 with the increasing bed capacity [19, 21]. It is an irony that despite high NMR in the country, the existing provincial service delivery standards have not yet defined the requirements for neonatologists and neonatal nurse specialists. For this study, and keeping in view the WHO and UNICEF standards for essential care services for small and sick NYIs [17, 20], the availability of the specialist workforce was assessed with at least one staff in these cadres at inpatient care settings. Our study findings highlighted the shortage of specialist staff (neonatologists, neonatal surgeons, and neonatal nurse specialists) mainly at DHQs. Our findings regarding the shortage of skilled newborn care are comparable to the studies conducted in other LMICs [30, 32, 33]. These findings are also consistent with previous literature suggesting that an inadequate number of trained and competent staff is a major challenge in NICUs across Pakistan [34]. The scarcity of the specialist staff in neonatal care at the district level could be attributed to the inadequacy in the sanctioned positions (especially, neonatologists and neonatal surgeons) at the health care facilities and inadequate financial resources.

A further challenge was insufficient attention to the capacity building of staff through continuing education. This was particularly seen in areas of parents’ counseling on infant death and advanced care for small and sick newborns. Similar findings have also been reported from previous studies in Pakistan and other countries, highlighting the lack of staff training as a serious concern [35, 36]. The underlying reasons could be inadequate resource allocation for staff training at the provincial and district health department. Our findings depicted that majority (61%) of the in-patient care facilities had conducted capacity building of staff in neonatal resuscitation. A similar observation has been recorded from an earlier study in the local context with a majority (80%) of the public health facilities equipped with neonatal resuscitation service (95% C.I. 69.0–88.0) [37]. However, these factors need to be explored further, and specialized neonatal care training of existing paediatricians and nurses needs to be planned and implemented accordingly if good quality NYIs care is to be provided.

Our study also assessed four facility management practices for NYIs care. This includes external supervision, management team meetings, interdisciplinary team meetings, and budgetary flexibility. With an exception of budgetary flexibility reported by the majority (57%) of the facilities, in general, very few facilities met the criteria for the remaining three components under facility management practices. Our findings indicated that very few facilities had management (17%) and interdisciplinary team meetings (30%) to discuss progress related to sick NYIs care. Interdisciplinary team meetings among neonatal care specialists (paediatricians, obstetricians, radiologists, nursing specialists, and pharmacists) are essential to improve coordination, identify needs, and enhance the plan of care for any particular patient [38]. This was a major gap identified by the study, which was predominantly due to the low priority given to such activities, and the excessive flow of patients in the public health care facilities.

Improvement in the care of sick NYIs in inpatient care settings requires the availability of the robust quality of care measures and indicators. It was appreciable to note that majority (70%) of the surveyed facilities monitored the quality of care indicators related to NYIs, however, very few (35%) facilities reported monitoring of nosocomial infections for NYIs. This presumably indicates less attention on preventing hospital-acquired infections and the absence of a surveillance mechanism to track nosocomial infection among NYIs at the surveyed health care facilities. Earlier studies have emphasized continuous monitoring and surveillance of infection rates among NYIs to prevent nosocomial infections [39]. Our results also indicated that most of the facilities (87%) had services in place to diagnose and treat specific conditions for sick newborns. Also, essential interventions for sick newborns were reported to be available from more than two-thirds of the surveyed facilities. Likewise, an earlier study in the context of emergency newborn care services in a province in Pakistan indicated that most (92%) of the public sector facilities had availability of parenteral antibiotics 90%, (95% C.I. 80.0–95.0) [37]. However, basic interventions for the newborn were reported from less than half of the facilities (43%) across the country. The availability of basic interventions such as thermal management, feeding and lactation support, and infection prevention practices (handwashing and practice of keeping one infant in a cot) are essential to safeguard a newborn’s health around the time of birth in inpatient care settings.

Alongside essential services and interventions for NYIs in inpatient care settings, discharge planning, and support to parents is also crucial safeguard health of sick children after discharge. Our findings illustrated that just over 50% of the facilities reported systems in place for adequate discharge planning. Whereas, most of the facilities fell short of developing linkages with community-based workers (13%) and strategies to promote adherence after discharge (35%).

Clinical documentation for sick NYIs is significant, as it provides an assessment of their condition on regular basis. Most of the reviewed records had captured information on vital signs and birth weight, however, information on danger signs and APGAR scoring remained undocumented in most of the NYIs records. An earlier study from Pakistan also shows that staff working in the NICUs are inadequately trained to recognize danger signs, which might be an obstacle for them to record the required information [40]. The identified gaps in the reporting of important indicators for newborns' health could be ascribed to the absence of a standardized mechanism of documentation at SNCUs across the secondary and tertiary care hospitals in Pakistan.

While clinical documentation for sick NYIs is important to track their progress, reliable and timely data on neonatal mortality and stillbirth is required to implement and evaluate health interventions and monitor the progress. Our findings showed that the routine information system in the surveyed hospitals lacked a unified mechanism to report data on neonatal deaths and stillbirths. Likewise, improvement in reporting neonatal deaths in Jordanian Hospitals has also been documented for tracking progress and taking appropriate actions to save sick newborns [41].

In general, our findings resonate with the observations reported from earlier studies that also highlighted gaps in service provision for inpatient neonatal care services [11]. There is a dire need to strengthen and increase the essential services for newborns to address the major causes of newborn morbidity and mortality in Pakistan i.e., prematurity (39%), birth asphyxia (21%), and, neonatal sepsis (17%) [42]. Pakistan also reports the highest proportion of children born with low birth weight, with 20.9% of newborns weighing below 2500 g [43]. This further accentuates the necessity for essential interventions for small and sick newborns, alongside increase coverage for KMC units.

The major strength of this cross-sectional survey was the assessment of health care facilities in the public health sector across the entire country with a focus on inpatient newborn care, which is the first of its kind at the national level. Second, we used standardized tools to collect information about inpatient NYI care services across public health care facilities. The use of purposive sampling was the major limitation of the study, alongside the absence of health facilities providing similar care in the private sector.

## Conclusions

The study has demonstrated important gaps towards improving the quality of newborn care in the inpatient settings in public hospitals of the country. This includes a shortage of KMC units, inadequate corrective and preventive maintenance and insufficient availability of essential drugs, a dearth of specialized neonatal care workforce, inadequacy in staff training, poor attention on inter-disciplinary meetings, insufficient attention on monitoring nosocomial infection, low availability of basic interventions for the newborn care, inadequate documentation of the important parameters for neonatal health, and inadequate attention on discharge planning and support for NYIs.

Eleven actions are recommended to ensure service readiness and to enhance the quality of inpatient NYIs care. These include – (i) Establishment of KMC units at the district and provincial hospitals with adequate supplies and equipment; (ii) Integration of KMC units across NICUs and SNCUs, (iii) Develop an action plan for the preventive and corrective maintenance of equipment, (iv) Ensure adequate availability of essential drugs, (v) Deploy specialized staff at DHQ hospitals that include neonatologists, neonatal surgeons, and neonatal nurses, (vi) Develop compulsory post-training service commitments with SNCUs across public sector hospitals, (vii) Mandatory set up of inter-disciplinary teams for improved coordination of care at inpatient care units, (viii) Build on the job capacity of health care providers to provide them with hands-on skills in neonatal care management, (ix) Ensure vigilant monitoring system to track nosocomial infections among NYIs, (x) Strengthen a standardized reporting format to capture information on important parameters of newborn care and a unified information system to track neonatal deaths and stillbirths in the hospital, and (xi) Reinforce discharge planning and support by developing linkages with community based workers and staff training to promote adherence of NYIs caretakers after discharge.

## Data Availability

The datasets used and/or analyzed during the current study are available from the corresponding author on reasonable request.
